# Definitions of recovery and reintegration across the first year: A qualitative study of perspectives of persons with spinal cord injury and caregivers

**DOI:** 10.1038/s41393-024-00962-1

**Published:** 2024-02-13

**Authors:** Kim D. Anderson, Anne M. Bryden, Brian Gran, Susan W. Hinze, Mary Ann Richmond

**Affiliations:** 1 Department of Physical Medicine and Rehabilitation, Case Western Reserve University School of Medicine, Cleveland, OH USA; 2 MetroHealth Center for Rehabilitation Research, MetroHealth System, Cleveland, OH USA; 3 Department of Sociology, Case Western Reserve University College of Arts and Sciences, Cleveland, OH USA; 4 Spinal Cord Injury/Disorders Center, Veteran Affairs Northeast Ohio Healthcare System, Cleveland, OH USA

**Keywords:** Spinal cord diseases, Outcomes research

## Abstract

**Study Design:**

Longitudinal, qualitative cohort study.

**Objectives:**

To understand how people with newly acquired spinal cord injury (PWS) and their support person (SP) define recovery and successful community reintegration (CR) across the first 12 months post-injury (mpi) and their satisfaction with the rate of recovery and reintegration experienced.

**Setting:**

Academic and Veterans hospitals in Midwest USA.

**Methods:**

In-depth, semi-structured interviews were conducted in two cohorts of PWS and SP during the initial inpatient rehabilitation stay, at 6 mpi, and at 12 mpi. Recordings were transcribed; four authors independently undertook line-by-line coding. The team discussed codes to reach consensus and synthesize into broader themes within the International Classification of Function, Disability, and Health and Transformative frameworks.

**Results:**

Data are reported on 23 PWS and 21 SP. PWS and SP are similar in defining recovery as gaining motor function and achieving independence. However, SP more frequently define recovery in terms of maintaining positivity and emotional recovery. At 12 mpi both groups shift to define recovery according to progress. Social roles, being active, and employment are persistent themes of how PWS and SP define successful CR. However, SP also frequently define successful CR as reestablishing identity and emotional adjustment. Veterans with SCI less frequently defined successful CR as employment.

**Conclusions:**

This study is the first to reveal how PWS and SP define recovery and reintegration during the first 12 mpi. Given decreasing lengths of stay, this information can be used to tailor rehabilitation strategies during the critical first year of injury to optimize recovery.

## Introduction

Spinal cord injury (SCI) affects multiple body systems as well as multiple aspects of life. The first 12 months post-injury (mpi) are exceptionally challenging and filled with many changes. Healthcare providers often discuss recovery from SCI in terms of neurologic recovery, with particular emphasis on prediction of motor and sensory recovery based on the International Standards for Neurologic Classification of SCI [1, 2]. There is a growing body of literature describing priorities for recovery from the perspectives of people living with SCI, but the majority of those data are from individuals more than 12 mpi. These priorities repeatedly involve gaining improvement in arm function, autonomic functions (bladder, bowel, sexual), reducing pain or spasticity, and varying degrees of walking [3, 4]. However, a gap remains in our understanding of how people with SCI define recovery during the first 12 months of injury.

Successful community reintegration is defined as the resumption of culturally and developmentally appropriate social roles after injury and is the goal of rehabilitation [5, 6]. Multiple barriers to successful community reintegration exist, however, and interventions are limited [7]. While tools have been created to measure degrees of community reintegration (Reintegration to Normal Living Index [5], Craig Handicap Assessment and Reporting Technique [8], Community Integration Measure [9]), these tools were not developed with input from people living with SCI. Indeed, the authors can find no published data on how people living with SCI define successful community reintegration.

To explore how recovery and community reintegration after SCI are experienced, it is necessary to use multi-faceted frameworks that reflect both the physical aspects of recovery as well as the social structures within which recovery experiences are rooted. The theoretical perspectives on which this study is based are a hybrid of the International Classification of Functioning, Disability and Health (ICF) [10] and the Transformative Framework [11]. A key feature of the ICF is its emphasis on positive language such as health and function, implicitly removing the deviance label from disability. The ICF incorporates social participation and recognizes the influence of personal and environmental factors to health, ensuring a comprehensive conceptualization of disability. While the ICF assists in linking functions of the body to activity capacity and performance and ultimately to participation in society, the Transformative Framework offers a critical lens to examine the socio-structural influences on people with disability who are living in an ableist world. The assumption underlying the Transformative Framework is that knowledge is not neutral. Knowledge is influenced by human interests and reflects the power and social structures within society [11]. Where the intent is to use knowledge to improve society, segments of society remain excluded. People with SCI, and disability in general, are often marginalized and not able to attain equal status with health professionals in determining their own care [12].

The objectives of this study were to (1) learn how people living with SCI define recovery and successful community reintegration across the first 12 months of injury and (2) understand their satisfaction with their recovery and reintegration experience. Due to the increasingly complicated healthcare and social services environments, support persons often take on significant roles helping their loved one with SCI navigate these systems while trying to achieve recovery and reintegration. Therefore, a third objective was to understand how support persons define recovery and successful community reintegration across the first 12 months of their loved one’s experience.

## Methods

### Research design

A multi-methods approach utilizing a combination of semi-structured interviews and validated outcome measures was employed in this longitudinal study. The qualitative interviews were comprehensive and explored individual definitions, perceptions, and expectations of recovery, including physical feelings, emotional responses, social support, and interpretation of the pace and timing of changes experienced, as well as barriers or facilitators they experienced in making decisions about treatment options or clinical trial participation and how their experience of recovery influenced their community reintegration. The current paper only presents results related to definitions of recovery and community reintegration and corresponding satisfaction at the end of the first 12 mpi.

### Participants

A criterion-based purposive sampling technique was used to recruit participants. Criterion-based purposive sampling is a non-probability sampling technique that allows recruitment of individuals based on specific criteria identified at the discretion of the investigators. Eligible participants were individuals 18 years or older with newly acquired spinal damage (any cause, level, and severity) participating in their initial inpatient rehabilitation stay or were the support person designated by the individual. All attempts were made to enroll equal numbers with tetraplegia and paraplegia, all AIS grades (A-D), various ages, and both sexes to ensure diverse perspectives would be obtained. Participants with SCI and their support persons were recruited from the inpatient SCI rehabilitation unit of a Veterans Affairs SCI Hub or an academic medical center in the Midwest United States (US). The goal was to enroll 15 people with SCI (PWS) and 15 support persons (SP) at each hospital for a total of 60 participants.

### Semi-structured interviews

A series of three in-depth semi-structured interviews were conducted with each participant over the first year after injury (one during inpatient rehabilitation, one approximately 6 mpi, and one at 12 mpi). An interview guide was used to ensure uniformity in data collection. The interview questions were open-ended and grounded in the ICF and Transformative frameworks and focused on multiple aspects of recovery and community reintegration. Interviews with PWS and SP were conducted separately whenever possible and typically lasted 30–60 minutes. Two team members were assigned to a PWS-SP dyad, and they conducted each of the interviews for that dyad.

### Outcome measures

In addition to the semi-structured interviews, three validated outcome measures were collected for the PWS. The International SCI Core Dataset version 2.0 [13] was collected by chart review of the inpatient rehabilitation stay to obtain data on length of hospitalization, cause of injury, level and severity of injury, and place of discharge. At the same time as the inpatient interview and 12-month interview, the Spinal Cord Independence Measure version III (SCIM III) [14] and Moorong Self-Efficacy Scale (MSES) [15] questionnaires were completed by interview format as gross measures of functional change and self-efficacy change across the first 12 mpi. SCI characteristics data are included in Table 1 while SCIM III and MSES data are included in Supplementary Tables 1-3.

**Table 1:** Demographics.

Variable	Persons with SCI (PWS) *N* = 23		Support Persons (SP) *N* = 21	
	Civilian (*n* = 16)	Veteran (*n* = 7)	Civilian (*n* = 16)	Veteran (*n* = 5)
**Sex** [*n* (%)]				
Male	12 (75)	7 (100)	1 (6)	0 (0)
Female	4 (25)	0 (0)	15 (94)	5 (100)
**Age at Injury** [mean years ± SD]	41 ± 18	52 ± 20	47 ± 16	53 ± 17
**Race** [*n* (%)]				
American Indian or Alaska Native	1 (6)	0 (0)	1 (6)	0 (0)
Asian	0 (0)	0 (0)	0 (0)	0 (0)
Black or African American	7 (44)	1 (14)	6 (38)	2 (40)
Native Hawaiian or Other Pacific Islander	0 (0)	0 (0)	0 (0)	0 (0)
White	8 (50)	6 (86)	9 (56)	3 (60)
**Injury level at Discharge** [*n* (%)]				
Tetraplegia	8 (50)	6 (86)	N/A	N/A
Paraplegia	8 (50)	1 (14)		
**AIS Grade at Discharge** [*n* (%)]				
A	5 (31)	1 (14)		
B	3 (19)	2 (29)	N/A	N/A
C	4 (25)	1 (14)		
D	4 (25)	3 (43)		
**Cause of SCI** [*n* (%)]				
Fall	4 (25)	2 (29)		
Assault	6 (38)	1 (14)		
Transport	1 (6)	2 (29)		
Sports	1 (6)	0 (0)		
Other traumatic	0 (0)	1 (14)	N/A	N/A
Vascular	1 (6)	0 (0)		
Infection	1 (6)	0 (0)		
Degenerative non-traumatic	1 (6)	0 (0)		
Other non-traumatic	0 (0)	1 (14)		
Unknown	1 (6)	0 (0)		
**Relation to PWS** [*n* (%)]				
Grandparent			1 (6)	0 (0)
Parent			5 (31)	0 (0)
Spouse			3 (19)	4 (80)
Domestic partner	N/A	N/A	3 (19)	0 (0)
Sibling			1 (6)	0 (0)
Child (adult)			2 (13)	1 (20)
Friend			1 (6)	0 (0)
**Timing of Interviews** [mean dpi ± SD]				
1st interview	42 ± 16	79 ± 27	46 ± 17	90 ± 25
2nd interview	197 ± 16	198 ± 13	198 ± 14	200 ± 12
3rd interview	372 ± 17	372 ± 5	376 ± 18	372 ± 7

*SCI* spinal cord injury, *PWS* persons with SCI, *SP* support persons; *SD* standard deviation; *AIS* American Spinal Injury Association Impairment Scale, *DPI* days post-injury.

### Data analysis

Interviews were recorded using a digital audio recorder. Each recording was transcribed verbatim by a professional with training and experience in transcription (Rev.com, San Francisco, CA USA), then checked for accuracy and deidentification by study staff. Deidentified transcripts were entered into the NVivo data management software program for organization and analysis. Transcripts were first coded independently by four authors (KDA, AMB, BG, SH), then consensus was reached on codes during group coding review sessions. Themes and sub-themes were generated using a constructivist grounded theory [16] analytic approach until theoretical saturation [17]. This method of critical inquiry acknowledges the active role of the researcher in constructing, shaping, and interpreting data and relies on deep reflexivity to examine assumptions, biases, and preconceptions throughout the research process [18]. Verification checks and feedback were obtained from multiple discussions with our community partner, the local chapter of the United Spinal Association, to ensure credible data.

## Results

### Participants

A total of 44 participants (people with SCI as well as support persons) provided informed consent and were enrolled from January 2020 through June 2022. At the academic medical center, 94 inpatients with SCI were screened between January 2020 through May 2021. Of those 94, 11 could not be approached during the COVID-19 lockdown of the unit, 22 did not qualify, 19 were discharged before consent could be assessed, 13 were not interested in participating, 10 with tetraplegia were not approached because of trying to enroll equal numbers with paraplegia, 2 were on the unit when enrollment was completed so were not approached, and 17 consented to the study. At the VA hospital, 43 inpatients with SCI were screened between April 2020 through June 2022. Of those 43, 6 could not be approached during the COVID-19 lockdown of the unit, 17 did not qualify, 13 were not interested in participating, and 7 consented to the study. Demographic information and timing of interviews for all participants is shown in Table 1. Most interviews were conducted over the telephone due to the COVID-19 pandemic. Regulatory restrictions at the Veteran Affairs hospital, related to the COVID-19 pandemic, impacted the ability to meet the target enrollment of Veterans with SCI and their support persons. However, theoretical saturation of themes was reached during the iterative analysis process.

### Definitions of recovery

**Fig. 1:**
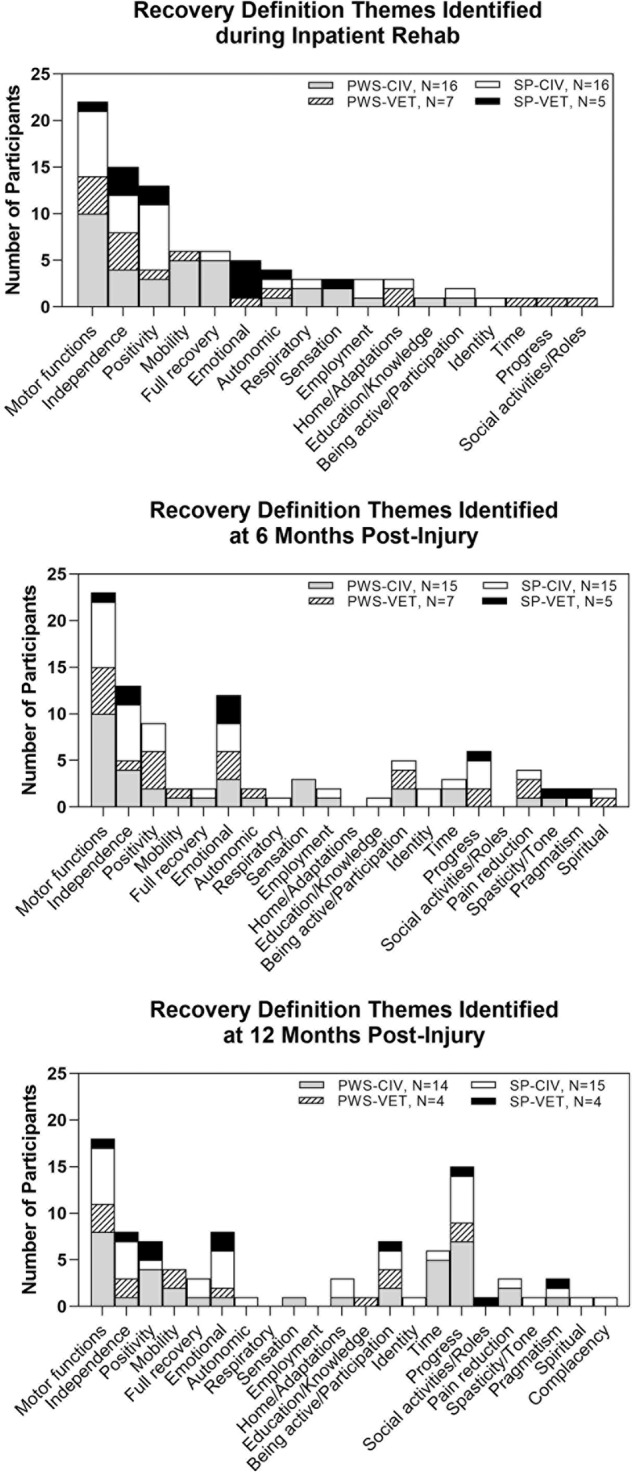
Frequency of recovery definition themes. **A** Themes identified during the inpatient rehabilitation interview. **B** Themes identified during the 6-month post-injury interview. **C** Themes identified during the 12-month post-injury interview. Frequency was based on the number of participants that identified each theme at a particular interview and was categorized by PWS civilian or Veteran and SP civilian or Veteran. Each participant may have defined recovery as more than one theme.

At each of the three interview time points (during inpatient rehabilitation stay, 6 mpi, and 12 mpi) participants were asked the question “*When you think about recovery after SCI, what does recovery mean to you (*Support Person *‘with respect to your loved one’)?”*. Twenty-two themes were generated from 122 transcripts. Table 2 lists each of the themes with excerpts from quotes representing each theme as well as the number of PWS and SP that identified each theme in his/her definitions of recovery. The five most common definitions of recovery involved gaining incremental motor functions, establishing independence, maintaining positivity, seeing progress, and making emotional recovery. Figure 1 illustrates how each of the four groups (PWS Civ, PWS Vet, SP Civ, SP Vet) defined recovery (frequency of themes), how the frequency of those definitions changed across the first 12 mpi, and when new definition themes emerged. Gaining motor functions was a persistent recovery theme across all time points; however, at 6 mpi, there was an increase in the frequency of defining recovery in terms of emotional recovery as well as an emergence of additional recovery themes involving pain reduction, spasticity/tone reduction, pragmatism, and spirituality. At 12 mpi defining recovery in terms of progress and time became predominant. For individual data on recovery definitions at each time point see Supplemental Table 1.

**Table 2:** Themes – Definitions of Recovery.

Themes	Representative Quotes
Motor functions (19 PWS, 16 SP)	*Use of my hands, raise my arms, improving balance, core strength, standing, eventually be walking, upper body strength, stronger leg muscles, core strength and stability of posture, movement in hands*
Independence (12 PWS, 14 SP)	*Able to do for myself, be self-sustainable, regain independence, be less dependent, take care of self, live independently, not have my wife do everything for me, do more by herself, build up independence*
Positivity (11 PWS, 9 SP)	*Take one day at a time, try my best, motivate not to give up, survive, be positive, uplift him as he deals with his injury, living each day as it comes, one step at a time, staying focused, get through this*
Progress (10 PWS, 7 SP)	*Came a long way, getting better, made some progress, continued improvement, he’s comin’ through, progressin’ a little bit, doin’ a lot better, better than I thought he would be already, mastering each stage of progress*
Emotional (6 PWS, 9 SP)	*Get stronger mentally, gain confidence to do more, deal with the new normal, find his own happiness, be there emotionally for him, have a strong mindset, mind over matter, learning to live with my limitations*
Being active/Participation (8 PWS, 4 SP)	*Get outside, get out of house and go places, enjoy life, being able to drive, be active, learn how to drive again, get out and go see people, be outside, drive again, hanging out with friends more*
Time (8 PWS, 2 SP)	*Slow, slow process, ongoing process, it’s a process, slowly getting better, take it slowly, slow but making headway, long and hard, slow*
Full recovery (7 PWS, 2 SP)	*100% functionality, everything working like before, getting back to before hurt, recover and be a success story, be functional like before, getting better and back to how his life used to be, 100% healed, make a full recovery*
Mobility (8 PWS, 0 SP)	*Be mobile, being able to move, gain mobility, lose weight, manage transferring, regain movement, be more mobile, transferring, exercise for strength and agility*
Autonomic (4 PWS, 3 SP)	*Go to the bathroom without a tube, poop on his own, bladder and bowel function, get bowel control back, biggest issue I’ve got is bowel care, the urinary*
Pain reduction (5 PWS, 2 PWS)	*To manage pain, reduce pain, less pain, pain management and how I can support that, alleviate pain in arms, still have pain in my arm, pain in my head, neck, and shoulders*
Home/Adaptations (3 PWS, 3 SP)	*Go home, get out of nursing home, move around the house and outside, get power wheelchair, get hospital bed, getting him home, going home*
Sensation (4 PWS, 1 SP)	*Feeling in legs, sensation in fingers, sensation down to legs, get feeling back in his legs, lower body sensation, leg sensation, sensation*
Employment (2 PWS, 3 SP)	*Return to work, return to his career, pursue in her career, generate income, working or school*
Pragmatism (1 PWS, 4 SP)	*Realistic and reachable goals, keeping focused on realistic goals, it’s gonna be just like it is now, his blood sugar stays well and he eats okay and he stays clean and has no skin breakdown*
Respiratory (2 PWS, 2 SP)	*Improve my speech, remove trach’, get off ventilator, get trach’ hole completely closed*
Education/Knowledge (2 PWS, 1 SP)	*Working or school, further my education, find out what’s going on inside of my neck*
Spasticity/Tone reduction (1 PWS, 2 SP)	*Tightness in my left hand and the tightness in my ankle, move her arms down for her because they keep rising up from the contracture, he’s not moving enough to make the stiffness go away*
Spiritual (1 PWS, 2 SP)	*Be a praying young lady, keeping faith in God, learn how to trust God, I’m sure his faith is rocked so he has to recover from that as well*
Identity (0 PWS, 3 SP)	*Get back to herself, be the same person he was, see him blossom back to his self, have her back to what/who she was*
Social activities/Roles (1 PWS, 1 SP)	*Interact with my grandchildren, join into more activities to be around more people*
Complacency (0 PWS, 1 SP)	*Can’t get him interested in much*

Numbers in parentheses indicate the number of participants that identified the theme.

*PWS* persons with spinal cord injury; *SP* support persons.

### Definitions of successful community reintegration

**Fig. 2:**
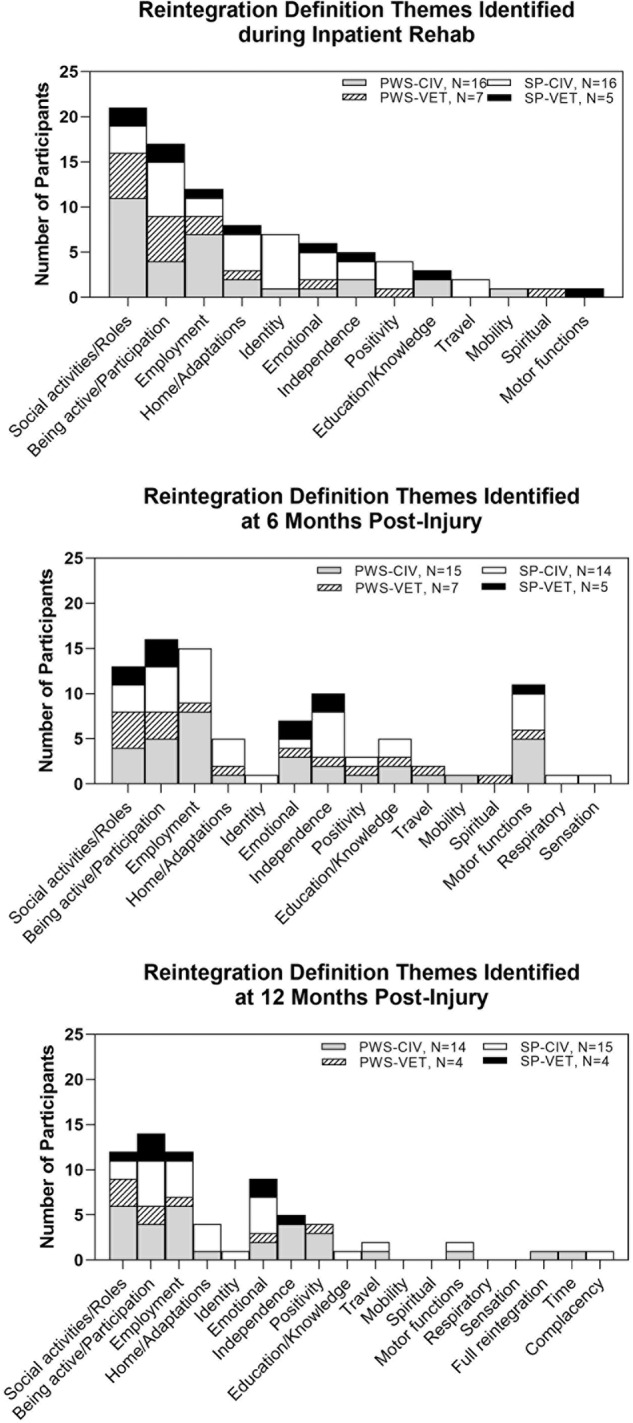
Frequency of successful community reintegration themes. **A** Themes identified during the inpatient rehabilitation interview. **B** Themes identified during the 6-month post-injury interview. **C** Themes identified during the 12-month post-injury interview. Frequency was based on the number of participants that identified each theme at a particular interview and was categorized by PWS civilian or Veteran and SP civilian or Veteran. Each participant may have defined successful community reintegration as more than one theme.

During each interview all participants were asked “*How would you define success* (Support Person – *‘of your loved one’) at getting back into life?*”. The phrase ‘getting back into life’ was used as more understandable terminology for community reintegration. Eighteen themes were generated from 121 transcripts. Table 3 lists each of the themes with excerpts from quotes representing each theme as well as the number of PWS and SP that identified each theme in his/her definitions of recovery. The five most common definitions of successful community reintegration involved returning to social activities and roles, being active/participating, pursuing employment, establishing independence, and emotionally adjusting to a new life. Figure 2 illustrates how each of the four groups (PWS Civ, PWS Vet, SP Civ, SP Vet) defined successful community reintegration (frequency of themes), how the frequency of those definitions changed across the first 12 mpi, and when new definition themes emerged. Returning to social activities and roles, being active/participating, and pursuing employment were persistent reintegration themes across all time points. At 6 mpi there was an increase in the frequency of defining successful reintegration in terms of regaining motor functions and establishing independence. At 12 mpi defining successful reintegration in terms of emotionally adjusting to a new life became a predominant theme. For individual data on reintegration definitions at each time point see Supplemental Table 2.

**Table 3:** Themes – Definitions of Successful Community Reintegration.

Themes	Representative Quotes
Social activities/Roles (20 PWS, 9 SP)	*Go to church, grocery shop with my wife, seeing my friends, take care of the children, walk my daughter down the aisle at her wedding, helping and inspiring other women, watch my son play* [sport]*, go to restaurants*
Being active/Participation (14 PWS, 15 SP)	*Cook, tinker around my farm, go fishing, mow my yard, get back on my bike, drive again, watch sports, get back to her regular doing, write a book about depression and coping, able to accomplish things and have purpose*
Employment (13 PWS, 10 SP)	*Get back to work, looking into work, pursue employment ideas, get a job, provide for my children financially, investigate work options, return to work in a new role, start a business or something*
Independence (8 PWS, 10 SP)	*Manage myself by myself, handle my business, manage and be independent, take care of himself, doing things on his own without assistance, go huntin’ on my own, use cell phones, tablets, and computers on my own*
Emotional (6 PWS, 10 SP)	*Learn to accept his situation, adjusting to new lifestyle based on level of recovery, do better mentally, trying to find this new normal however he determines that, I’m a success right now - I’m livin’, healing right*
Motor functions (7 PWS, 7 SP)	*Use hands, walk, able to climb up and down, improve hand function, use cane instead of walker, strengthen hands, walking again, walk up and down stairs without fear of falling, get her hands to function*
Home/Adaptations (5 PWS, 7 SP)	*Modify house to go home, get out of house by himself, set up technology in house so she has more independence, modify house so he can get in more rooms and do more by himself, live together again, come home*
Positivity (6 PWS, 4 SP)	*Stay positive, feel supported, support my son’s definition of success, getting through the day, pressing forward through each day, doin’ the best I can, I gotta have a goal, if not I could just lay in bed and say ‘well this is it’*
Education/Knowledge (5 PWS, 3 SP)	*Going back to school, talk with therapist to help know what to do, more education, working with voc rehab to get some training with a more not physically based job, search YouTube for reputable medical lectures on my injury*
Travel (3 PWS, 2 SP)	*Travel, day trips, little vacation trips, travel*
Identity (1 PWS, 3 SP)	*Back to being herself, bring him more back to life, express herself and go forth, have a good lifestyle and own property*
Mobility (2 PWS, 0 SP)	*Having mobility, be mobile*
Spiritual (1 PWS, 0 SP)	*Trusting in God, telling’ everyone of God’s goodness*
Time (1 PWS, 0 SP)	*Slowly but surely everything takes time*
Full reintegration (1 PWS, 0 SP)	*Being able to function the way I did before*
Complacency (0 PWS, 1 SP)	*It’s nothing changed right now*
Sensation (0 PWS, 1 SP)	*Feel fingers*
Respiratory (0 PWS, 1 SP)	*Get trach hole completely closed*

Numbers in parentheses indicate the number of participants that identified the theme.

*PWS* persons with spinal cord injury, *SP* support persons.

### Satisfaction with recovery and community reintegration

At the third interview time point (12 mpi) participants were asked the following questions:

- “*How satisfied are you with your (*Support Person *‘your loved one’s) rate of recovery over this first year?”*.
- “*How complete do you feel your (*Support Person *‘your loved one’s) recovery is?”*.
- “*How satisfied are you with (*Support Person *‘your loved one’s) ‘getting back into life’ over this first year?”*.

Quotes describing satisfaction with rates of recovery and community reintegration fell into low, medium, or high levels of satisfaction. Table 4 lists representative quotes for each level of satisfaction. Of the 18 PWS and 19 SP interviewed at 12 mpi, 3 PWS and 3 SP felt their recovery was complete or almost complete, 12 PWS and 16 SP felt their recovery was not complete, 2 PWS were unsure, and 1 PWS did not view recovery in those terms. For individual quotes on complete recovery, satisfaction with rate of recovery, and satisfaction with rate of community reintegration see Supplemental Table 3.

**Table 4:** Levels of satisfaction with recovery and community reintegration.

Satisfaction with rate of recovery over first 12 months	
Low	*- Not satisfied at all*. *- Well, pretty low. I’m not, I’m not totally satisfied*. *- 30% satisfied*.
Medium	*- Satisfied, but not too satisfied. I did wish it was more, but I gotta be patient*. *- I mean, there’s a lot more I can do, but I’m satisfied with my recovery up to this point. I wish it was better*. *- Um, you know, I wish it was better, but, uh, I’ve noticed some improvements in leg movements and my therapists have also said they notice stuff. Um, so I guess, uh, I’m, uh, happy with it, but I could be happier*.
High	*- Oh, very satisfied. Very satisfied. Yeah. Absolutely. Because a year ago, I- I was convinced I’d never be able to walk again*. *- Uh, in the whole first year, I’ve really, excuse me. I’d have to say, like, 80%*. *- Um, I’m pre- I’m pretty satisfied. ‘Cause, you know, I- I’m able to do a lot more on my own, or that I know how to do on my own*.
**Satisfaction with community reintegration over first 12 months**	
Low	*- Um, I can be a lot better. I could be a lot more satisfied, but it’s just … You know, I get frustrated because I can’t do what I used to do*. *- Um, not that good…Uh, I just wish I can get out more, and just be around more…* *- I’m not satisfi- I don’t like to talk about that, too much.…Because it keeps me… It, it takes my mind off my short term. My short term goals, which…you know, I had different goals before. But-…to answer your question, I, I’m not too happy with it*.
Medium	*- During the summer, great. During the winter, not so much*. *- Oh, not bad. I mean, I really haven’t… You know, I’m retired, so I don’t have to work…And, uh, and with the COVID, we don’t go out much anyhow… You know, so…* *- Uh, satisfied. I am, uh, I’m encouraged…To keep… Yeah, I’m encouraged to keep moving for some reason, and I, and that, that creates excitement inside of me*.
High	*- I’m pretty well satisfied…Yup. I’m pre- pretty well satisfied*. *- Oh, um, very s- satisfied…Um, yeah. I was even able to find an apartment that was accessible, so…Well, that’s a task, I found out*. *- I’m definitely satisfied with it. Uh, being able to, you know, be included with my friends in things, and get, you know, going out a little bit more, at least*

## Discussion

These are the first data to report how people living with SCI and their support persons define recovery and successful community reintegration early after injury onset and to describe how those definitions change across the first 12 months. This sheds light into the real-world experience of sustaining a life changing injury like SCI and how navigating through healthcare systems and social structures while trying to recover and reintegrate influences those definitions. The ICF is a relevant framework to support how PWS and SP define recovery and successful community reintegration. The Transformative framework is relevant in supporting the satisfaction/dissatisfaction in attaining recovery and reintegration during the first 12 months because it incorporates how social structures, knowledge, and advocacy influence the success of recovery and reintegration.

PWS (regardless of civilian or Veteran status) typically defined recovery as gaining motor function and achieving independence, but changes to those definitions emerged over time. For example, the increase in the frequency of defining recovery in terms of emotional recovery at 6 months could be related to experiencing the challenging transition from rehabilitation to home or rehabilitation to skilled nursing facility to home [19]. Additionally, this finding may be related to the varying experiences of psychological and social adjustment to SCI [20, 21]. Similarly, emerging recovery definitions of pain reduction and spasticity/tone reduction at 6 months could be related to the manifestation of those conditions after people leave short rehabilitation stays [22]. Managing those conditions can be difficult if access to SCI-specialized care is lost once in the community. Defining recovery in pragmatic or spiritual terms emerged at 6 months for a small number of PWS. This could be due to understanding one’s limitations, whether personal or environmental, or looking to higher powers outside of oneself to make sense of one’s injury [23, 24].

Support persons, however, more frequently defined recovery in terms of maintaining positivity and emotional recovery. This may not be surprising on a surface level as the role of a support person is to provide support. When one cannot provide physical support, it is natural to try to provide emotional support. While health professionals are necessary to achieve physical recovery and *can* provide social support, designated support persons are better positioned to provide emotional support after a traumatic injury. Women have typically provided this support for kin with medical needs, and our data are consistent with this gendered pattern. Women were named as the support person in 20 of the 21 cases and included mothers, wives, girlfriends, daughters, a sister, a grandmother, and a female friend. We only had 1 male support person (a husband) and, though he never defined recovery in terms of positivity or emotional, we cannot make comparisons to understand if male support persons are as likely as women to report positivity and emotional recovery as significant.

At 12 months this study found a strong shift by both people with SCI and support persons to define recovery in terms of time and progress. The prominent emergence of these themes raises an interesting question. Do healthcare providers create pressure or a sense of a ‘deadline’ when discussing prognosis of recovery? It is known that on average most of the motor and sensory recovery occurs within the first 6 to 12 months post-injury [for review see [2]], but that does not mean recovery cannot occur at later times [25]. There are various clinical prediction tools healthcare providers can use to help guide prognosis [26], but these are based on probabilities and cannot identify a unique individuals’ recovery path. When told that most of one’s recovery will occur in the first year, it is understandable that time and progress, or lack of progress, may begin to influence how people view their recovery. But how can recovery potential be maximized in a healthcare system and social structure fraught with barriers that limit access to interventions that could enhance recovery during that crucial first 12 months of an injury? Future publications will present data collected as part of this larger study on barriers and facilitators people experience while seeking recovery across the first 12 months post-injury. Those data may provide explanation for the data presented here regarding mixed satisfaction in the rate of recovery as well as the majority of PWS and SP feeling recovery was not complete at 12 months.

It is not surprising that regaining social roles, being active, and pursuing employment were persistently strong components of how people with SCI and support persons define successful reintegration. It is interesting that at 6 months there is a surge in defining successful reintegration around regaining motor functions and independence. This finding may reflect dissatisfaction with the rate of recovery of motor functions and independence. It may also reflect the relationship between physical recovery/functional independence and interacting with the community environment. Short inpatient rehabilitation stays not only limit the maximization of interventions that can promote recovery, but also limit the ability to prepare people with SCI and support persons for the difficult transition back to the community [19]. Slow gains in physical recovery or independence could negatively influence an individual’s ability to be active in the community [22], hence influence how they define successful community reintegration. Interestingly, Veterans with SCI less frequently defined successful community reintegration in terms of employment. This could reflect the age difference (Table 1) between the Veterans with SCI in this study compared to civilians, though this study was not designed to be quantitative. Some Veterans may also receive financial support based on their service history that could reduce the need to think about employment. Support persons also frequently defined successful community reintegration as reestablishing identity and emotional adjustment. There are data supportive of this concept from individuals with acquired brain injury indicating that they feel reestablishing identity is important to the recovery process [27].

### Limitations

As with any study, there are limitations. Most notably, the findings must be interpreted within the context of the COVID-19 pandemic. Interviews started two months before the nationwide lockdown in 2020 and concluded in early 2023 before the pandemic was officially declared as over. All participants’ experiences during the first 12 months post-injury were impacted by the pandemic in some way or another. A recent study demonstrated that people with new SCI as well as their caregivers experienced challenges while trying to transition from inpatient rehabilitation to home during the pandemic [28]. We have captured additional data from our study that will be presented in future publications. Another limitation is that all participants were from one geographic area in the Midwest US. Experiences in other regions may be different. However, experiences were captured from two different healthcare systems, a Veteran Affairs (VA) healthcare system and a private sector healthcare system. The VA healthcare system is an interconnected system of care that focuses on systemwide clinical expertise and a team-based approach that incorporates physical, psychosocial, and economic determinants of health for both the Veteran and family members/caregivers [29]. This has many similarities to national healthcare systems in other countries. The private healthcare sector in the US simply cannot, or will not, provide that level of care uniformly to everyone. Every attempt was made to ensure a diverse sample of participants and, thus, a diverse sample of perspectives. There were more traumatic causes of SCI compared to non-traumatic causes (78% vs 22%, respectively). The civilian sample of PWS was relatively similar to the US national traumatic SCI statistics with respect to age, sex, cause, level, and severity. There were no participants of Hispanic ethnicity, however, and there was an over-representation of non-Hispanic Black participants that mirrors the regional population. The Veteran sample of PWS did not have female or Hispanic representation and had an over-representation of individuals with incomplete tetraplegia. The civilian and Veteran samples of SP were disproportionately female. Finally, due to the pandemic, fewer Veterans were enrolled in this study than civilians. As a result, this may have amplified or suppressed potential differences between Veterans and civilians. Though this study was not powered to be quantitative, there were trends suggesting demographic differences between the Veterans and civilians that were enrolled.

## Conclusion

As a result of the decreasing length of stay for rehabilitation in the US, there is a need to tailor interventions offered early after injury toward the perspectives of those living the injury experience, both people with SCI and their support persons. In addition, rehabilitation healthcare providers could provide information prior to discharge about a variety of interventions tailored to the individuals’ desires to seek out in the community. These two strategies could enhance access to interventions important to recovery and reintegration during the critical first year post-injury. Future publications from this study will shed light on the experience of seeking out interventions to promote recovery and reintegration and the barriers and facilitators experienced while trying to access those interventions.

### Supplementary information

Supplementary Material

## Data Availability

A significant amount of data are included in Supplementary Tables 1-3. Additional data are available upon request from the corresponding author.
