# Supplementary material for: Definitions of recovery and reintegration across the first year: A qualitative study of perspectives of persons with spinal cord injury and caregivers
Source: Spinal Cord. 2024 Feb 13;62(4):156–63. doi: 10.1038/s41393-024-00962-1 (PMC11003865; doi:10.1038/s41393-024-00962-1)
Supplement: Supplementary file 1 — Supplementary Material [file 41393_2024_962_MOESM1_ESM.pdf]

Supplemental Table 1. Individual recovery definition themes over time are provided along with individual data regarding injury, sex, age, race, function (SCIM III), and self-efficacy (MSES).

Participant	Inpatient rehabilitation	6 months post-injury	12 months post-injury
PWS-Civ-101<br>**C4 AIS C; M; 54; B/AA; SCIM ∆ -6; MSES ∆ 7**	Employment; Respiratory; Mobility	Motor functions	Time; Pragmatism; Mobility; Home/Adaptation
PWS-Civ-102<br>**C7 AIS A; M; 36; W; SCIM ∆ 1; MSES ∆ -15**	Independence; Motor functions; Sensation	Independence; Sensation	Time; Positivity; Motor functions
PWS-Civ-103<br>**C4 AIS A; F; 65; W; SCIM ∆ 5; MSES ∆ 2**	Motor Functions; Respiratory	Motor functions	Time; Independence; Motor functions; Pain
PWS-Civ-104<br>**T7 AIS A; M; 73; W; SCIM ∆ -4; MSES ∆ -10**	Mobility; Independence; Motor functions; Sensation	Sensation; Autonomic	Positivity; Be active/Participation
PWS-Civ-105<br>**C4 AIS B; M; 28; B/AA; SCIM ∆ 0; MSES ∆ -25**	Mobility; Motor functions; Full recovery; Independence	Independence; Mobility; Employment	Progress; Motor functions
PWS-Civ-106<br>**C6 AIS D; F; 69; W; SCIM ∆ 53; MSES ∆ 21**	Motor functions	Motor functions; Be active/Participation; Sensation	Full recovery
PWS-Civ-107<br>**T6 AIS C; M; 29; W; SCIM & MSES ∆ N/A**	Independence	LOST	LOST
PWS-Civ-108<br>**T11 AIS B; M; 56; W; SCIM ∆ 29; MSES ∆ -1**	Motor functions; Autonomic	Full recovery; Motor functions	Progress; Motor functions
PWS-Civ-109<br>**C4 AIS B; F; 25; B/AA; SCIM ∆ 14; MSES ∆ 23**	Full recovery; Mobility	Motor functions; Emotional; Independence	Independence; Progress
PWS-Civ-110<br>**C5 AIS D; F; 21; B/AA; SCIM & MSES ∆ N/A**	Full recovery; Mobility; Be active/Participation	Emotional; Independence	LOST
PWS-Civ-111<br>**C8 AIS D; M; 22; B/AA; SCIM ∆ 29; MSES ∆ 8**	Full recovery; Education	Spasticity/Tone; Motor functions	Progress; Motor functions; Sensation
PWS-Civ-112<br>**T12 AIS D; M; 42; B/AA; SCIM ∆ 33; MSES ∆ -4**	Full recovery; Motor functions	Positivity; Motor functions	Progress; Pain reduction; Positivity; Time
PWS-Civ-113<br>**T10 AIS A; M; 38; B/AA; SCIM ∆ 10; MSES ∆ 27**	Positivity	Time; Emotional; Pain reduction	Positivity; Emotional

PWS-Civ-115<br>**L1 AIS C; M; 52; AN; SCIM ∆ 10; MSES ∆ 1**	Motor functions; Positivity	Motor functions; Be active/Participation	Time; Motor functions
PWS-Civ-116<br>**L1 AIS B; M; 27; W; SCIM ∆ 8; MSES ∆ 17**	Positivity; Motor functions	Motor functions	Progress; Motor functions; Mobility
PWS-Civ-117<br>**T9 AIS C; M; 18; W; SCIM ∆ 10; MSES ∆ 0**	Motor functions	Positivity; Time; Motor functions	Progress; Motor functions; Be active/Participation
PWS-Vet-101<br>**C6 AIS D; M; 22; W; SCIM & MSES ∆ N/A**	Motor functions; Progress; Independence	Progress; Motor functions	Withdrawn by PI
PWS-Vet-102<br>**C5 AIS C; M; 69; W; SCIM ∆ 16; MSES ∆ -2**	Home/Adaptations; Independence	Progress; Autonomic; Positivity	Progress; Motor functions
PWS-Vet-103<br>**T5 AIS A; M; 39; W; SCIM ∆ 10; MSES ∆ -9**	Positivity	Positivity; Pain reduction; Be active/Participation; Independence	Independence; Be active/Participation
PWS-Vet-104<br>**C4 AIS D; M; 71; B/AA; SCIM & MSES ∆ N/A**	Motor functions; Autonomic; Home/Adaptations; Spiritual	Emotional; Motor functions; Spiritual	Passed away
PWS-Vet-105<br>**C1 AIS D; M; 72; W; SCIM ∆ 0; MSES ∆ -17**	Motor functions; Social activities/Roles	Motor functions; Pain reduction; Mobility; Emotional	Emotional; Mobility; Be active/Participation; Motor functions; Education/Knowledge
PWS-Vet-106<br>**C7 AIS B; M; 37; W; SCIM & MSES ∆ N/A**	Time; Emotional; Independence	Motor functions; Emotional; Positivity	Withdrawn by PI
PWS-Vet-107<br>**C5 AIS B; M; 52; W; SCIM ∆ -11; MSES ∆ -11**	Motor functions; Mobility; Independence	Positivity; Motor functions; Be active/Participation	Progress; Motor functions; Mobility; Independence
SP-Civ-201<br>**F; 54; W**	Motor functions	Independence	Home/Adaptation
SP-Civ-202<br>**F; 35; W**	Motor functions; Positivity	Independence; Emotional; Identity	Emotional; Home/Adaptation
SP-Civ-203<br>**M; 66; W**	Missing	Be active/Participation; Independence; Respiratory	Independence; Motor functions; Pain; Be active/Participation; Spasticity/Tone
SP-Civ-204<br>**F; 71; W**	Motor functions	Motor functions	Complacency; Pragmatism
SP-Civ-205<br>**F; 47; B/AA**	Respiratory; Independence; Employment	Time; Pragmatism; Motor functions	Emotional; Independence; Spiritual

SP-Civ-206<br>**F; 69; W**	Independence	Motor functions; Emotional	Time; Motor functions
SP-Civ-207<br>**F; 36; W**	Motor functions	WITHDREW	WITHDREW
SP-Civ-208<br>**F; 24; W**	Positivity; Motor functions	Progress; Positivity	Autonomic; Progress
SP-Civ-209<br>**F; 47; B/AA**	Identity; Be active/Participation; Positivity	Progress; Independence	Progress; Emotional; Identity
SP-Civ-210<br>**F; 60; B/AA**	Independence; Employment; Motor functions	Emotional; Spiritual; Motor functions; Full recovery	Progress; Independence; Emotional
SP-Civ-211<br>**F; 38; B/AA**	Positivity	Identity; Positivity	Progress; Motor functions
SP-Civ-212<br>**F; 63; B/AA**	Full recovery	Progress; Motor functions	Full recovery
SP-Civ-213<br>**F; 28; B/AA**	Positivity	Positivity; Pain reduction	Positivity
SP-Civ-215<br>**F; 33; AN**	Independence; Positivity	Independence; Motor functions	Be active/Participation; Motor functions
SP-Civ-216<br>**F; 29; W**	Motor functions; Autonomic	Motor functions	Full recovery; Motor functions
SP-Civ-217<br>**F; 57; W**	Positivity; Home/Adaptations	Independence; Employment; Education/Knowledge	Motor functions; Progress; Independence
SP-Vet-201	Never enrolled		
SP-Vet-202<br>**F; 74; B/AA**	Emotional; Positivity	Progress; Independence; Emotional	Progress; Positivity
SP-Vet-203<br>**F; 34; W**	Emotional; Positivity; Independence	Emotional	Emotional; Positivity; Motor functions
SP-Vet-204<br>**F; 69; B/AA**	Independence	Spasticity/Tone reduction; Pragmatism	Did not query this topic
SP-Vet-205<br>**F; 44; W**	Emotional; Autonomic; Independence	Emotional	Emotional; Social activities/Roles
SP-Vet-206	Never enrolled		
SP-Vet-207<br>**F; 45; W**	Emotional; Sensation; Motor functions	Motor functions; Independence	Pragmatism; Be active/Participation; Independence

PWS-Civ – person with SCI, civilian; PSW-Vet – person with SCI, Veteran; SP-Civ – support person of a civilian; SP-Vet – support person of a Veteran; AIS – ASIA Impairment Scale; M – male; F – female; B/AA – black or African American; W – white; AN – Alaska Native; SCIM Δ – change in Spinal Cord Independence Measure III at 12 months post-injury (interview 3) from inpatient rehabilitation (interview 1); MSES Δ – change in Moorong Self-Efficacy Scale at 12 months post-injury (interview 3) from inpatient rehabilitation (interview 1); N/A – not available

Supplemental Table 2. Individual successful community reintegration definition themes over time with individual data regarding injury, sex, age, race, function (SCIM III), and self-efficacy (MSES).

**Participant**	**Inpatient rehabilitation**	**6 months post-injury**	**12 months post-injury**
PWS-Civ-101 **C4 AIS C; M; 54; B/AA; SCIM Δ -6; MSES Δ 7**	Employment; Social activities/roles; Be active/Participation	Employment; Be active/Participation; Motor functions	Independence; Home/Adaptation; Employment
PWS-Civ-102 **C7 AIS A; M; 36; W; SCIM Δ 1; MSES Δ -15**	Home/Adaptations; Independence	Home/Adaptation	Be active/Participation; Social activities/roles
PWS-Civ-103 **C4 AIS A; F; 65; W; SCIM Δ 5; MSES Δ 2**	Social activities/roles	Motor functions	Full reintegration; Travel; Be active/Participation; Independence
PWS-Civ-104 **T7 AIS A; M; 73; W; SCIM Δ -4; MSES Δ -10**	Be active/Participation; Social activities/roles	Motor functions	Be active/Participation; Social activities/roles
PWS-Civ-105 **C4 AIS B; M; 28; B/AA; SCIM Δ 0; MSES Δ -25**	Social activities/roles; Employment	Independence; Mobility; Employment	Independence
PWS-Civ-106 **C6 AIS D; F; 69; W; SCIM Δ 53; MSES Δ 21**	Employment; Social activities/roles	Employment; Be active/Participation; Social activities/roles	Employment; Be active/Participation; Motor functions
PWS-Civ-107 **T6 AIS C; M; 29; W; SCIM & MSES Δ N/A**	Home/Adaptations; Employment; Social activities/roles	LOST	LOST
PWS-Civ-108 **T11 AIS B; M; 56; W; SCIM Δ 29; MSES Δ -1**	Employment	Employment; Motor functions	Emotional; Employment
PWS-Civ-109 **C4 AIS B; F; 25; B/AA; SCIM Δ 14; MSES Δ 23**	Social activities/roles	Be active/Participation; Employment; Social activities/roles; Independence	Emotional; Social activities/Roles
PWS-Civ-110 **C5 AIS D; F; 21; B/AA; SCIM & MSES Δ N/A**	Education/Knowledge; Social activities/roles	Social activities/roles; Be active/Participation; Travel	LOST
PWS-Civ-111 **C8 AIS D; M; 22; B/AA; SCIM Δ 29; MSES Δ 8**	Employment; Identity	Emotional; Employment; Education/Knowledge	Employment; Independence; Social activities/Roles
PWS-Civ-112 **T12 AIS D; M; 42; B/AA; SCIM Δ 33; MSES Δ -4**	Employment; Social activities/roles	Emotional; Social activities/roles	Positivity; Employment; Social activities/Roles
PWS-Civ-113	Mobility; Emotional	Emotional; Positivity	Positivity

**T10 AIS A; M; 38; B/AA; SCIM ∆ 10; MSES ∆ 27**			
PWS-Civ-115<br>**L1 AIS C; M; 52; AN; SCIM ∆ 10; MSES ∆ 1**	Education; Social activities/roles; Be active/Participation	Employment	Positivity
PWS-Civ-116<br>**L1 AIS B; M; 27; W; SCIM ∆ 8; MSES ∆ 17**	Independence	Education; Employment	Employment
PWS-Civ-117<br>**T9 AIS C; M; 18; W; SCIM ∆ 10; MSES ∆ 0**	Be active/Participation; Social activities/roles	Motor functions; Be active/Participation	Time; Social activities/Roles
PWS-Vet-101<br>**C6 AIS D; M; 22; W; SCIM & MSES ∆ N/A**	Be active/Participation; Social activities/Roles; Employment	Be active/Participation; Employment	Withdrawn by PI
PWS-Vet-102<br>**C5 AIS C; M; 69; W; SCIM ∆ 16; MSES ∆ -2**	Emotional; Home/Adaptations; Be active/Participation	Social activities/Roles; Emotional	Emotional; Positivity
PWS-Vet-103<br>**T5 AIS A; M; 39; W; SCIM ∆ 10; MSES ∆ -9**	Be active/Participation; Social activities/Roles	Independence; Be active/Participation	Social activities/Roles; Be active/Participation
PWS-Vet-104<br>**C4 AIS D; M; 71; B/AA; SCIM & MSES ∆ N/A**	Social activities/Roles; Positivity; Spiritual	Social activities/Roles; Motor functions; Spiritual	Passed away
PWS-Vet-105<br>**C1 AIS D; M; 72; W; SCIM ∆ 0; MSES ∆ -17**	Be active/Participation	Be active/Participation; Education/Knowledge	Social activities/Roles
PWS-Vet-106<br>**C7 AIS B; M; 37; W; SCIM & MSES ∆ N/A**	Social activities/Roles; Employment	Social activities/Roles; Home/Adaptations; Positivity	Withdrawn by PI
PWS-Vet-107<br>**C5 AIS B; M; 52; W; SCIM ∆ -11; MSES ∆ -11**	Be active/Participation; Social activities/Roles	Social activities/Roles; Travel	Be active/Participation; Employment; Social activities/Roles
SP-Civ-201<br>**F; 54; W**	Home/Adaptations	Home/Adaptation; Motor functions; Sensation	Emotional
SP-Civ-202<br>**F; 35; W**	Positivity	Home/Adaptation; Identity	Be active/Participation
SP-Civ-203<br>**M; 66; W**	Travel; Be active/Participation	Be active/Participation; Independence; Respiratory	Home/Adaptation; Emotional; Travel; Motor functions
SP-Civ-204<br>**F; 71; W**	Home/Adaptations; Travel; Be active/Participation; Independence	Home/Adaptation; Education/Knowledge	Home/Adaptation

SP-Civ-205<br>**F; 47; B/AA**	Home/Adaptations	Independence	Employment; Home/Adaptation
SP-Civ-206<br>**F; 69; W**	Employment; Social activities/roles	Employment; Motor functions; Be active/Participation; Social activities/roles	Social activities/roles
SP-Civ-207<br>**F; 36; W**	Independence; Be active/Participation; Positivity	WITHDREW	WITHDREW
SP-Civ-208<br>**F; 24; W**	Be active/Participation; Social activities/roles	Social activities/roles; Motor functions	Employment
SP-Civ-209<br>**F; 47; B/AA**	Identity; Social activities/roles	Be active/Participation; Emotional	Be active/Participation
SP-Civ-210<br>**F; 60; B/AA**	Missing	Missing	Emotional; Identity
SP-Civ-211<br>**F; 38; B/AA**	Positivity	Independence; Employment	Employment; Social activities/Roles
SP-Civ-212<br>**F; 63; B/AA**	Emotional	Independence; Employment; Motor functions	Complacency
SP-Civ-213<br>**F; 28; B/AA**	Emotional	Positivity; Social activities/roles; Employment	Emotional
SP-Civ-215<br>**F; 33; AN**	Be active/Participation	Employment; Independence	Be active/Participation; Employment
SP-Civ-216<br>**F; 29; W**	Emotional; Home/Adaptations	Be active/Participation; Employment	Be active/Participation
SP-Civ-217<br>**F; 57; W**	Be active/Participation; Employment	Be active/Participation; Education	Education; Be active/Participation
SP-Vet-201	Never enrolled		
SP-Vet-202<br>**F; 74; B/AA**	Motor functions; Be active/Participation	Independence; Be active/Participation	Emotional; Social activities/Roles; Be active/Participation
SP-Vet-203<br>**F; 34; W**	Education/Knowledge; Employment; Social activities/Roles	Emotional	Employment; Be active/Participation
SP-Vet-204<br>**F; 69; B/AA**	Independence	Motor functions; Social activities/Roles; Be active/Participation	Did not query this topic
SP-Vet-205<br>**F; 44; W**	Emotional	Emotional	Emotional; Be active/Participation
SP-Vet-206	Never enrolled		
SP-Vet-207<br>**F; 45; W**	Be active/Participation; Social activities/Roles; Home/Adaptations	Independence; Be active/Participation; Social activities/Roles	Independence

PWS-Civ – person with SCI, civilian; PSW-Vet – person with SCI, Veteran; SP-Civ – support person of a civilian; SP-Vet – support person of a Veteran; AIS – ASIA Impairment Scale; M – male; F – female; B/AA – black or African American; W – white; AN – Alaska Native; SCIM Δ – change in Spinal Cord Independence Measure III at 12 months post-injury (interview 3) from inpatient rehabilitation (interview 1); MSES Δ – change in Moorong Self-Efficacy Scale at 12 months post-injury (interview 3) from inpatient rehabilitation (interview 1); N/A – not available

Supplemental Table 3. Individual data on complete recovery satisfaction with recovery and reintegration along with individual data regarding injury, sex, age, race, function (SCIM III), and self-efficacy (MSES).

Participant	Satisfaction with Recovery Quote	Satisfaction level	Satisfaction with Reintegration Quote	Satisfaction level	Complete Recovery Quote
PWS-Civ-101 **C4 AIS C; M; 54; B/AA; SCIM ∆ -6; MSES ∆ 7**	*Not satisfied at all.*	Low	*I'm not satisfi- I don't like to talk about that, too much....Because it keeps me... It, it takes my mind off my short term. My short term goals, which...you know, I had different goals before. But-...to answer your question, I, I'm not too happy with it.*	Low	*Complete. Wow. Uh, not complete at all. I definitely expected to be home by now. Um, I definitely expected to be, uh, to be able to, uh, manage myself in a walker. Uh, I'm not there.*
PWS-Civ-102 **C7 AIS A; M; 36; W; SCIM ∆ 1; MSES ∆ -15**	*Uh, so so.*	Medium	*I, um... Well, again, the, reintegrating into the community is nearly impossible with social distancing and the masks and the limitations of places that are open, so it's, it's pretty difficult right now.*	Low	*No, I, no, I don't. I'm still hopeful on, uh, some improvement um, so I'm remaining hopeful.*
PWS-Civ-103 **C4 AIS A; F; 65; W; SCIM ∆ 5; MSES ∆ 2**	*Well, pretty low. I'm not, I'm not totally satisfied.*	Low	*Missing*	(Opinion of interviewers: Low)	*Well, I was going to ask you guys that because, I don't know. Is there any more room for recovery in my situation? Or am I at the end? I, I honestly don't know.*
PWS-Civ-104 **T7 AIS A; M; 73; W; SCIM ∆ -4; MSES ∆ -10**	*Oh, pretty good. I guess. You know, I realize I probably wasn't gonna get recovered on my movement stuff, but, I realize that now.*	High	*I'm pretty well satisfied…Yup. I'm pre- pretty well satisfied.*	High	*I'm fully... I think I'm fully recovered... the way I am.*
PWS-Civ-105 **C4 AIS B; M; 28; B/AA; SCIM ∆ 0; MSES ∆ -25**	*Satisfied, but not too satisfied. I did wish it was more, but I gotta be patient.*	Medium	*Um, not that good…Uh, I just wish I can get out more, and just be around more...*	Low	*Um, like 2 out of 10. I think I got a lot of recovery left…Uh... Go out into the world again, live regular, normal. Have fun. Stuff like that.*
PWS-Civ-106	*Oh, very satisfied. Very satisfied. Yeah. Absolutely. Because a year ago, I- I*	High	*I'm fine. I mean, very satisfied.*	High	*If we had to put a percentage on it, again I would say 80% because there are some things that I have*

Participant	Satisfaction with Recovery Quote	Satisfaction level	Satisfaction with Reintegration Quote	Satisfaction level	Complete Recovery Quote
**C6 AIS D; F; 69; W; SCIM ∆ 53; MSES ∆ 21**	*was convinced I'd never be able to walk again.*				*not recovered as fast as I would like them…*
PWS-Civ-107 **T6 AIS C; M; 29; W; SCIM & MSES ∆ N/A**	LOST TO FOLLOW UP				
PWS-Civ-108 **T11 AIS B; M; 56; W; SCIM ∆ 29; MSES ∆ -1**	*Well, considering how bad I was hurt, I'm doing great. Yeah, I'd like to be better, but I'm not, I have to deal with that you know? And, so life is what it is, you know? I'm just, I'm trying to learn to accept it, and it's tough.*	Medium	*Um, I can be a lot better. I could be a lot more satisfied, but it's just ... You know, I get frustrated because I can't do what I used to do. (laughs)…Oh it's just frustrating because I used to have these things I used to be able to do right away, and I can't do it no more.*	Low	*There's a lot, a lot of things missing…Well I can tell I don't have any control of my, my balance, and that's because I don't have any muscles in my butt…Those hasn't come back yet and hopefully when they do come back up, I'll be able to lose the cane and crutch…*
PWS-Civ-109 **C4 AIS B; F; 25; B/AA; SCIM ∆ 14; MSES ∆ 23**	*30% satisfied.*	Low	*Uh, how satisfied am I? What's the scale? [Interviewer: Whatever you want. One to a hundred.] …Um, oh, 55…There's more to go. Yeah. Yeah, there's more to go. But I'm in a good state of mind right now, so, you know, I don't know, I just feel good with everything right now…Oh no, I said 55, because you just- 'cause I'm- I'm- I'm halfway there.*	Medium	*Interviewers recall of participant words: she's still having- she needs to- to work more on her left hand, yes… she did feel that the pregnancy did, um, affect her recovery,…Yeah, she seemed to feel like, because she had an epidural, that might've slowed down her- her recovery, I believe in her left leg.*
PWS-Civ-110 **C5 AIS D; F; 21; B/AA; SCIM & MSES ∆ N/A**	LOST TO FOLLOW UP				

Participant	Satisfaction with Recovery Quote	Satisfaction level	Satisfaction with Reintegration Quote	Satisfaction level	Complete Recovery Quote
PWS-Civ-111 **C8 AIS D; M; 22; B/AA; SCIM ∆ 29; MSES ∆ 8**	*Uh, in the whole first year, I've really, excuse me. I'd have to say, like, 80%.*	High	*Uh, over the first year, I'd have to say satisfied. Uh, a lot of times, you know, just being a young man who's used to stuff like that, you know, in my 20s, so it's, like, you know, I wanna live on, God, do the things I did before…But, you know, with this whole life change, um, it- it put a stop to things and it did change my life for the better or worse, but it was a major change.*	Medium	*Uh, just about almost complete. Like, you know, some things that are there, but, um, not like how it used to be before, but I think that's just wanting to get improvement on that is all, but, um, like, I'm able to walk but, like, not, like, properly, you know, speed walk or run like I used to.*
PWS-Civ-112 **T12 AIS D; M; 42; B/AA; SCIM ∆ 33; MSES ∆ -4**	*What, from one to 10? About like a, uh, six.*	Medium	*Oh, uh, well, I, I'm really satisfied, because my brother, he be in my, in my corner, and my, uh, mother be helping me.*	High	*I mean, it's, it's coming along, but I still, I still got a long way to go, to me…I mean, I'm walking, um, a little bit better.*
PWS-Civ-113 **T10 AIS A; M; 38; B/AA; SCIM ∆ 10; MSES ∆ 27**	*I know as far as satisfaction with me, um, I, I will say that, um, I'm inspired to keep going.*	Medium	*Uh, satisfied. I am, uh, I'm encouraged…To keep... Yeah, I'm encouraged to keep moving for some reason, and I, and that, that creates excitement inside of me.*	Medium	*Um, again, that's a, it's a, it's a question that, uh, it's, it's, it's, it's not that it's difficult to answer, but it p-, it places the circumstance in the position of like a, like there's an agenda for achievement, and I don't look at it like that, you know?...*
PWS-Civ-115 **L1 AIS C; M; 52; AN; SCIM ∆ 10; MSES ∆ 1**	*I mean, there's a lot more I can do, but I'm satisfied with my recovery up to this point. I wish it was better.*	Medium	*Um... I'm fairly satisfied. I mean, I, I've got, I, I, uh, I'm okay with my recovery because I'm going forward, I'm getting better, I'm, I'm getting stronger. So yeah, I'm, I'm, I'm pretty satisfied…*	Medium	*Um, I feel I have, so, I feel I have more healing to do. Right now I'm just continuing to get stronger, um... mm. Mentally I'm just forging ahead. I mean, it's difficult, I've had good days, bad days, you know?*
PWS-Civ-116	*Um, you know, I wish it was better, but, uh, I've noticed some*	Medium	*Oh, um, very s- satisfied…Um, yeah. I was even able to find an apartment that was accessible,*	High	*Uh, I was gonna say, well, what I think is I think I'm still earlier in the recovery. Maybe, um, you*

Participant	Satisfaction with Recovery Quote	Satisfaction level	Satisfaction with Reintegration Quote	Satisfaction level	Complete Recovery Quote
**L1 AIS B; M; 27; W; SCIM Δ 8; MSES Δ 17**	*improvements in leg movements and my therapists have also said they notice stuff. Um, so I guess, uh, I'm, uh, happy with it, but I could be happier.*		*so...Well, that's a task, I found out. But... (laughs)...It's a...It's a hard task, but...*		*know, a lot of the doctors told me it could be two plus years before, uh, we know for sure if anything is, uh, permanent or not. So I think I'm fairly early slash halfway-ish, maybe (laughs) if that makes sense.*
PWS-Civ-117 **T9 AIS C; M; 18; W; SCIM Δ 10; MSES Δ 0**	*Uh, very satisfied.*	High	*I'm definitely satisfied with it. Uh, being able to, you know, be included with my friends in things, and get, you know, going out a little bit more, at least.*	High	*Um, there's still work to be done. It's always still work to be done though, so...I mean, my goal is to be as close to as normal as I was before. And I know it'll take time, but you know, I'm- I'm in it for the long run...*
PWS-Vet-101 **C6 AIS D; M; 22; W; SCIM & MSES Δ N/A**	Withdrawn by PI, SP never enrolled				
PWS-Vet-102 **C5 AIS C; M; 69; W; SCIM Δ 16; MSES Δ -2**	*Well, you know, I'm satisfied, but I think it's going slower than it should.*	Medium	*Oh, not bad. I mean, I really haven't... You know, I'm retired, so I don't have to work. ...And, uh, and with the COVID, we don't go out much anyhow...You know, st...You know, so... And, uh, like I say, I've got a deck on the back of the house, and, you know, you got fire pit and stuff, and brothers and sister come over and see me every so often, you know.*	Medium	*Oh, well, I got a ways to go yet, but I- I- I'd say I'm maybe 20% there...That's all I can say. It's... You know, to me it's slow, like I said. It... But, i- it's going good and I, I can see improvement every week or two, you know?*
PWS-Vet-103 **T5 AIS A; M; 39; W; SCIM Δ 10; MSES Δ -9**	*Um, I'm pre- I'm pretty satisfied. 'Cause, you know, I- I'm able to do a lot more on my own, or*	High	*I'm pretty satisfied.*	High	*I'd say- I'd say about 75%. [Interviewer: And what does- what does that mean you're missing? What's the other 25%*

Participant	Satisfaction with Recovery Quote	Satisfaction level	Satisfaction with Reintegration Quote	Satisfaction level	Complete Recovery Quote
	that I know how to do on my own.				*you'd like to see get back?]* *Transfers and being able to drive.*
PWS-Vet-104 **C4 AIS D; M; 71; B/AA; SCIM & MSES Δ N/A**	Passed away before final interview				
PWS-Vet-105 **C1 AIS D; M; 72; W; SCIM Δ 0; MSES Δ -17**	*It depends on what day of the week it is, seriously. [Interviewer: Okay. Tell me where you're at today.] Today, I'm not too bad. The weather's not causing any severe pain.*	Medium	*During the summer, great. During the winter, not so much. I have come to the conclusion, uh, I have that sad seasonal effect disorder-... and that drags me down. ... I'm starting to feel better.*	Medium	*Yes and no. I look at my life before I fell and broke my neck and I look at the limitations that I have now... since the fractures and I still am learning to cope with them...*
PWS-Vet-106 **C7 AIS B; M; 37; W; SCIM & MSES Δ N/A**	Withdrawn by PI, SP never enrolled				
PWS-Vet-107 **C5 AIS B; M; 52; W; SCIM Δ -11; MSES Δ -11**	*Oh, I don't know. It probably should be better, um, but, um, you know, one of the, I guess, setbacks is my weight. I'm a 'lil heavy and it makes it hard to do certain things.*	Medium	*Pretty good, I would say. I'm still unable to do a lot of things, but-I am able to do a lot more today than I was a year ago.*	High	*Oh, I mean, it's getting there. Uh, I can do quite a bit on my own, I guess. I guess the one thing that I'm lacking is the ability to drive the van. If I can just lose some weight, then I think I will be able to make further strides. Uh, especially like doing transfers again.*
SP-Civ-201 **F; 54; W**	*Well, that's obviously been very disappointing.*	Low	*Uh, it has- it hasn't been great. I mean, his- his smartphone that I forced him into getting has helped a lot...'Cause he's been able to keep in touch with people.*	Low	*Well, I don't think his recovery- his recovery has been complete. And, like-... I think what's really missing is me being there.*
SP-Civ-202 **F; 35; W**	*Um, you know, it, um, it's been rough on him, you know, 'cause of course,*	Low	*Uh, I'd say so so 'cause, you know, he's, I mean he's learning like there's some people that he*	Medium	*I mean, he's a little bit ... 'Cause yeah, no even back when he was... he was losing blood from*

Participant	Satisfaction with Recovery Quote	Satisfaction level	Satisfaction with Reintegration Quote	Satisfaction level	Complete Recovery Quote
	you know, he's been, you know, always been an independent person... and, you know, and, you know, there's things that you see in his face that, you know, it, it's hard, you know?		*used to hang out, he don't hang out with no more, which is not really, not a positive impact in his life, so, you know, it's, it's kinda like, you know, 50-50, you know.*		*somewhere-... so he went to the hospital. Um, you know, that kind of set him back a little bit…*
SP-Civ-203 **M; 66; W**	*Uh, it's, it's a lot slower than we would like…Like I said, we're just basically maintaining her.*	Low	Missing	Missing	*I don't feel it's come along very much at all, maybe 20%...We were told it would be like a marathon, a long you know road to travel, but, uh, you know, uh, a year over, a little over a year and it's, um, we just feel like we're languishing or just like, you know, um, kinda running in place…*
SP-Civ-204 **F; 71; W**	*Well, we were hoping for a little more movement but... with this damn virus and stuff, we just haven't been able to, you know, get out and do anything about it.*	Low	*Right now I could say I'm not.*	Low	*Probably about as complete as it can be.*
SP-Civ-205 **F; 47; B/AA**	Missing	Missing	*Oh, gosh. I am super satisfied. I mean, he's a fighter. That's my child.*	High	*Oh, there's much more to do.*
SP-Civ-206 **F; 69; W**	*Well putting the word rate aside and saying that she is doing, she has come a long way. I mean, it, it, it's wonderful. Um, I just, you know, I think it's slower than we've hoped for but, but I think that's based on, again, going back to not working at the strengthening*	High	*Oh, I'm very dissatisfied. She hasn't got, she has not gotten back into life, period.*	Low	*She does. I feel she does, a lot more.*

Participant	Satisfaction with Recovery Quote	Satisfaction level	Satisfaction with Reintegration Quote	Satisfaction level	Complete Recovery Quote
	exercises…But, um, she's, um, she's doing great. *[Interviewer: So there, I mean there's some degree of satisfaction with her recovery?]* *Absolutely*				
SP-Civ-207 **F; 36; W**	WITHDREW				
SP-Civ-208 **F; 24; W**	*I'm pretty, I'm honestly, I'm pretty impressed. Like yesterday, we were sitting at the table and he just has a lot more mobility.*	High	*I'm pretty satisfied. I've noticed a big change in his personality, just in his mental health.*	High	*At this point, I'd say about 65%.*
SP-Civ-209 **F; 47; B/AA**	Missing	Missing	Missing	Missing	*I give it a seven [out of 10].*
SP-Civ-210 **F; 60; B/AA**	*I'm very sat- I'm very satisfied, because it has been improvement, it has been effort on her part, it has been determination, and, uh, uh, she, she always keep a smile on her face.*	High	Missing	Missing	*Uh, I, I, I don't feel like there's anything missing.*
SP-Civ-211 **F; 38; B/AA**	*Blown away. I'm... very satisfied.*	High	*Um, 100% satisfied with that.*	High	*We're almost at the top of the mark for 100%.*
SP-Civ-212 **F; 63; B/AA**	*Um ... I mean, I'm getting there. I m- I mean I ain't 100%, you know, thrilling about ... I mean, but ... Well, well, well not ... It's not so much the recovery, it's just that, I mean, he just still s- stuck. He, he's not getting no help.*	Medium	*I give it a half and half. That's all I can do…'Cause I'm not gonna think of the worst and I'm never gonna say he ... Oh, he ain't gonna better or ju- I don't know. I'm not gonna think that way, because I'll be thinking long.*	Medium	*It's, it's, um, 30% because like I say, he ain't ... I mean, he, he ... he might be healed mentally but physically he's not healed at all.*
SP-Civ-213 **F; 28; B/AA**	*But also now seeing how, uh, much progress he's also made, um, in terms of*	High	*Um, I'm f- I'm pretty satisfied. I, I think for me, you know, watching him having to, uh,*	High	*Well, I mean, I, with spinal cord injuries from what I've learned is we don't know. I mean,*

Participant	Satisfaction with Recovery Quote	Satisfaction level	Satisfaction with Reintegration Quote	Satisfaction level	Complete Recovery Quote
	pulling forward, um, since that first day, um, it's just been pretty amazing.		*you know, go through this drastic change, um, because he was pretty much, you know, independent and...Um, and so just to see him get back to, you know, things that he used to do, it, it makes me happy...*		*something, uh, miraculous can happen tomorrow. And, um, I feel that there's still more for him in terms of just his, um, his mobility, um, is concerned*
SP-Civ-215 **F; 33; AN**	*Um, really satisfied. Um, I kinda wanted to do, like, a, like, a progress party, which is kind of, like, you know, it's been a year now. But I mean, it's kind of weird to do, like, a- a anniversary of a tragic accident. You know what I mean? But- but- but his recovery that he's had so far has been so great to us that, um, you know, I really would like to celebrate his progress.*	High	*I'm satisfied enough that I'm so excited to, you know, like, put together clips of all of his (laughs) uh, therapies that I took videos of, and you know-... and to see that progress. You know, I'm- I'm very satisfied with where he's come so far, and, um, you know, but I- I just... I want to see him there. You know, I want to see him like I said, you know, in a job and in his truck and- and to be fully himself again.*	Medium	*He's- he's definitely, he's not all the way there.*
SP-Civ-216 **F; 29; W**	*Um I think, a lot has happened in the last year like he still has a lot to work on but...Things have gotten better since we - from where we started.*	Medium	*Um, I'm pretty satisfied with that. I mean like I said, like I think he's been successful at doing those things so therefore, I'm satisfied with them.*	High	*Um, I don't think it's - maybe like halfway complete? Like he's gotten more feeling, he's moving his one leg a lot more than the other. But he's moving a lot like in aquatherapy, um, so. I mean he's not all the way there yet but, better.*
SP-Civ-217 **F; 57; W**	*I am, I mean, I think we're amazed and we've, you know, uh, just when we were told, when we left rehab, that he would never walk again ... um,*	High	*Fantastic. I mean, his, his mental attitude has just been, um, everything, but po-, you know, everything positive. There's no ... I mean, sure, there are times that he was just frustrated-... but there was*	High	*We're not complete yet. He's got ... he's, he's continuing to improve every week. So, I mean, I, I don't think there's a limit yet of what his recovery will be.*

Participant	Satisfaction with Recovery Quote	Satisfaction level	Satisfaction with Reintegration Quote	Satisfaction level	Complete Recovery Quote
	this is phenomenal, absolutely phenomenal.		*never depression. There was never, um, a quitting. There was never, you know, any of that, that, that bogged him down.*		
SP-Vet-201	Never enrolled				
SP-Vet-202 **F; 74; B/AA**	*Oh, I- I'm- I'm- I'm very satisfied. Not satisfied to the point that, you know, that's it. But satisfied as far as up to this point.*	High	*Oh, I'm satisfied. Yeah.*	High	*I- I would say like, uh, probably 60.*
SP-Vet-203 **F; 34; W**	*Um, I'm very impre- impressed actually. I know it's not, it's not easy, and, you know, we've go- we've had some hiccups along the way.*	High	*So far, really good. Um, I know it hasn't been easy, and I've seen it, the not easy parts. Mm-hmm. But he still somehow pushes past it.*	High	*Um, hm. I would say, like 75, 80 percent I would say.*
SP-Vet-204 **F; 69; B/AA**	Did not query this topic				
SP-Vet-205 **F; 44; W**	*Um, I guess I would say good. It's not something I've ever prepared or expected-... like this is a, a take it day by day kind of thing...So, I have nothing to compare it to. So, I think it's good.*	Medium	*I have been very satisfied.*	High	*Physically, I think it's as good as, it's complete physically. I think he's, he's gotten to the point where he needs to be. But emotionally, mentally, his, his confidence levels, I don't know if, uh, if that's complete. I don't know. I think there could be more.*
SP-Vet-206	Never enrolled				
SP-Vet-207 **F; 45; W**	*Um, I probably would say not very happy, but I don't know, necessarily, if that's anybody's fault but our own.*	Low	*I think over this last six months it really has, um, increased. I'm pretty happy with it. The first six months, I think he was still kind of in shock. He really didn't wanna leave the house.*	High	*Oh, I- I think we're just at the ... honestly, I just think we're at the infantile stages now. Now- now that a lot of doors have opened up, I think- I think 107 will be able to recover so much faster because the acceptance ... of needing to recover is there now.*

PWS-Civ – person with SCI, civilian; PSW-Vet – person with SCI, Veteran; SP-Civ – support person of a civilian; SP-Vet – support person of a Veteran; AIS – ASIA Impairment Scale; M – male; F – female; B/AA – black or African American; W – white; AN – Alaska Native; SCIM Δ – change in Spinal Cord Independence Measure III at 12 months post-injury (interview 3) from inpatient rehabilitation (interview 1); MSES Δ – change in Moorong Self-Efficacy Scale at 12 months post-injury (interview 3) from inpatient rehabilitation (interview 1); N/A – not available
